# Supplementary material for: Towards annual updating of forced warming to date and constrained climate projections
Source: Nat Commun. 2025 Oct 17;16:9214. doi: 10.1038/s41467-025-63026-9 (PMC12534604; doi:10.1038/s41467-025-63026-9)
Supplement: Supplementary file 1 — Supplementary Information [file 41467_2025_63026_MOESM1_ESM.pdf]

Towards annual updating of forced warming to date and constrained climate projections

Supplementary Information

**Criteria / estimator →** <br>**Model ↓**	bias		std		RMSE	
	1-y	10-y	1-y	10-y	1-y	10-y
ACCESS-ESM1-5	0.040	-0.076	0.053	0.028	0.066	0.081
CNRM-ESM2-1	-0.046	-0.163	0.103	0.090	0.112	0.186
CanESM5	-0.223	-0.366	0.041	0.048	0.227	0.370
IPSL-CM6A-LR	-0.008	-0.133	0.047	0.056	0.047	0.144
MIROC-ES2L	-0.042	-0.133	0.028	0.029	0.050	0.136
MIROC6	0.017	-0.067	0.033	0.042	0.037	0.078
MPI-ESM1-2-LR	0.025	-0.058	0.026	0.035	0.036	0.068
UKESM1-0-LL	-0.083	-0.241	0.046	0.058	0.094	0.248
**Multi-model mean**	**-0.040**	**-0.155**	**0.052**	**0.052**	**0.103**	**0.190**
**Multi-model median**	**-0.025**	**-0.133**	**0.0435**	**0.045**	**0.058**	**0.14**

Table S1: Statistical properties (bias, standard deviation, root mean square error) of the 1-yr and 10-yr estimators of the GST forced response (see description in main text), for a subset of 8 CMIP6 models. The calculation is made for one SSP2-4.5 simulation for each model, and results are averaged over the period 2015-2100. The reference used is an estimate of the (true) forced response based on all available members and a filtering procedure (see Methods).

Criteria / estimator → <br>Model ↓	bias		std		RMSE	
	1-y	10-y	1-y	10-y	1-y	10-y
ACCESS-ESM1-5	-0.103	-0.249	0.094	0.094	0.207	0.285
CNRM-ESM2-1	0.075	-0.039	0.168	0.151	0.287	0.249
CanESM5	-0.045	-0.217	0.099	0.116	0.155	0.256
IPSL-CM6A-LR	-0.064	-0.206	0.107	0.092	0.212	0.234
MIROC-ES2L	-0.026	-0.135	0.104	0.106	0.126	0.175
MIROC6	-0.082	-0.173	0.074	0.075	0.139	0.197
MPI-ESM1-2-LR	0.003	-0.080	0.098	0.093	0.125	0.139
UKESM1-0-LL	-0.277	-0.465	0.153	0.162	0.332	0.499
**Multi-model mean**	**-0.065**	**-0.195**	**0.121**	**0.119**	**0.224**	**0.296**
**Multi-model median**	**-0.048**	**-0.178**	**0.102**	**0.112**	**0.165**	**0.218**

Table S2: Statistical properties (bias, standard deviation, root mean square error) of the 1-yr and 10-yr estimators of the regional (France) forced response (see description in main text), for a subset of 8 CMIP6 models. The calculation is made for four SSP2-4.5 simulations for each model. Results are averaged over simulations and over the period 2015-2100. The reference used is an estimate of the (true) forced response based on all available members and a filtering procedure (see Methods).

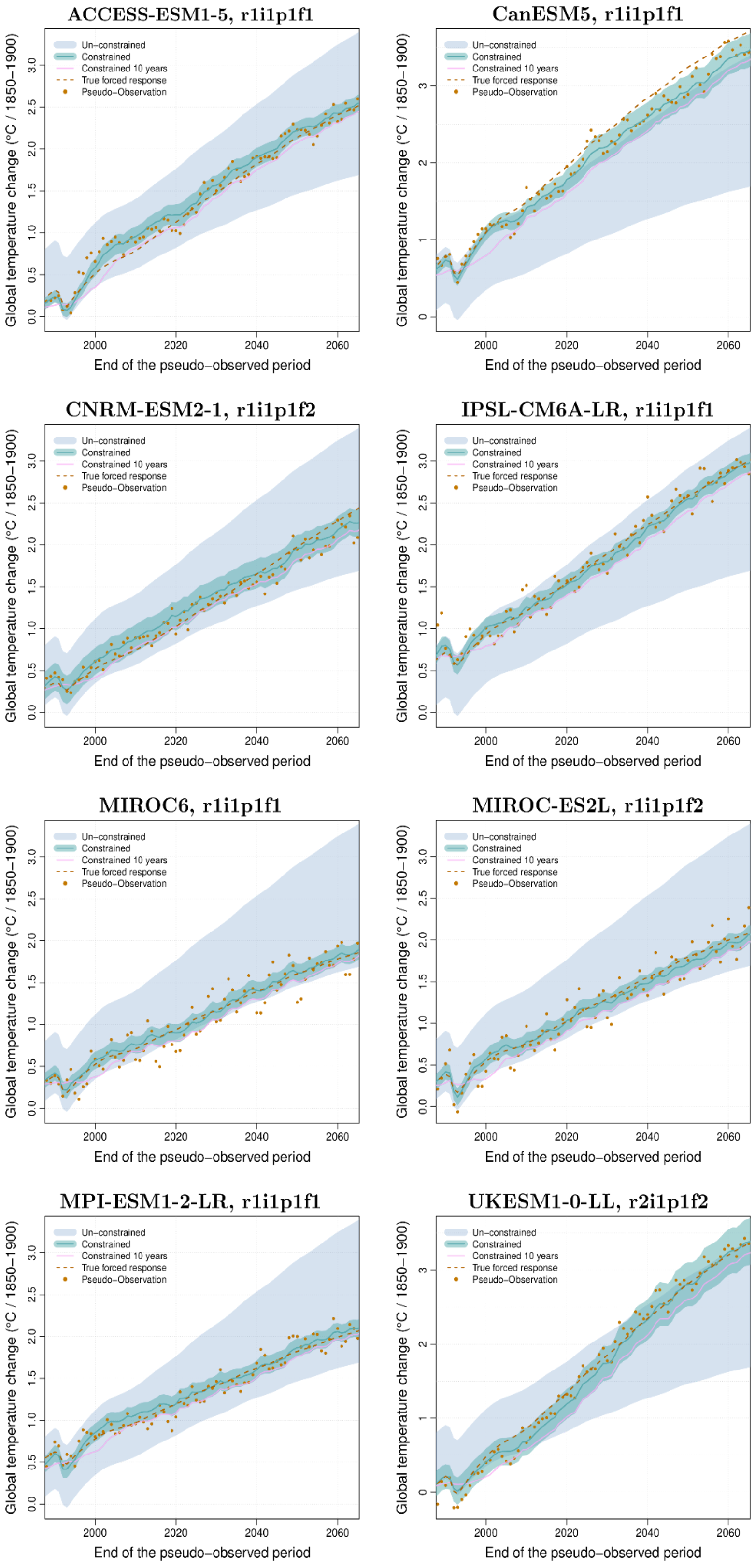

Figure S1: Same as Figure 2a, but using GST data from different CMIP6 models as pseudo-observations. This analysis provides an additional test of the method used to estimate the forced warming to date. It illustrates the properties of the 1-yr and 10-yr estimators, as compared to the estimated *true* forced response.

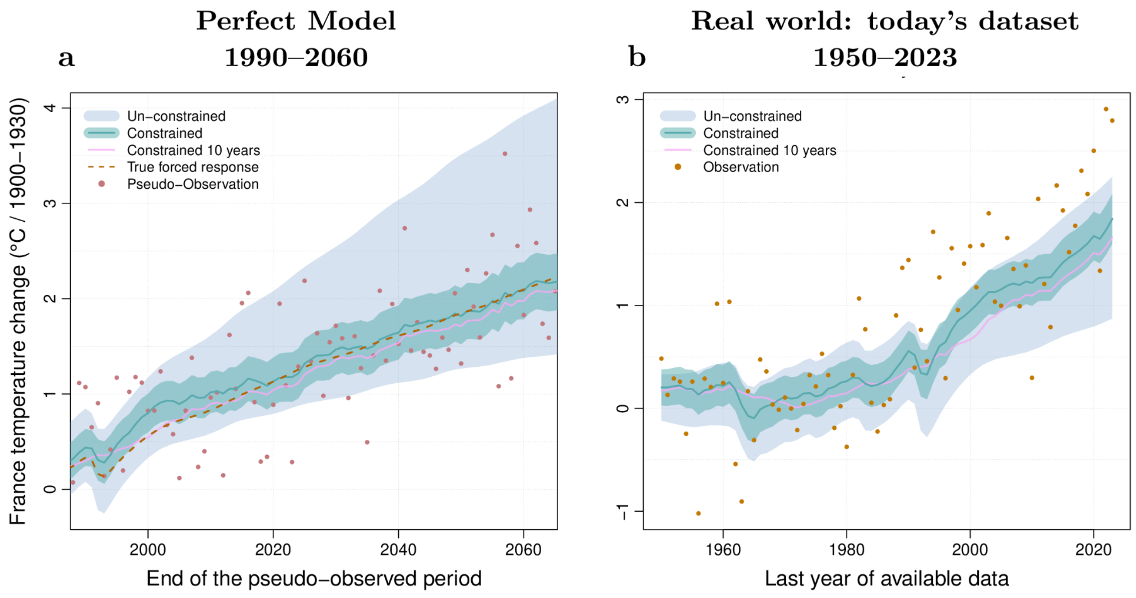

Fig S2: Same as Figure 2 (panels a and b only), for regional temperature (mean temperature over France), instead of GST.

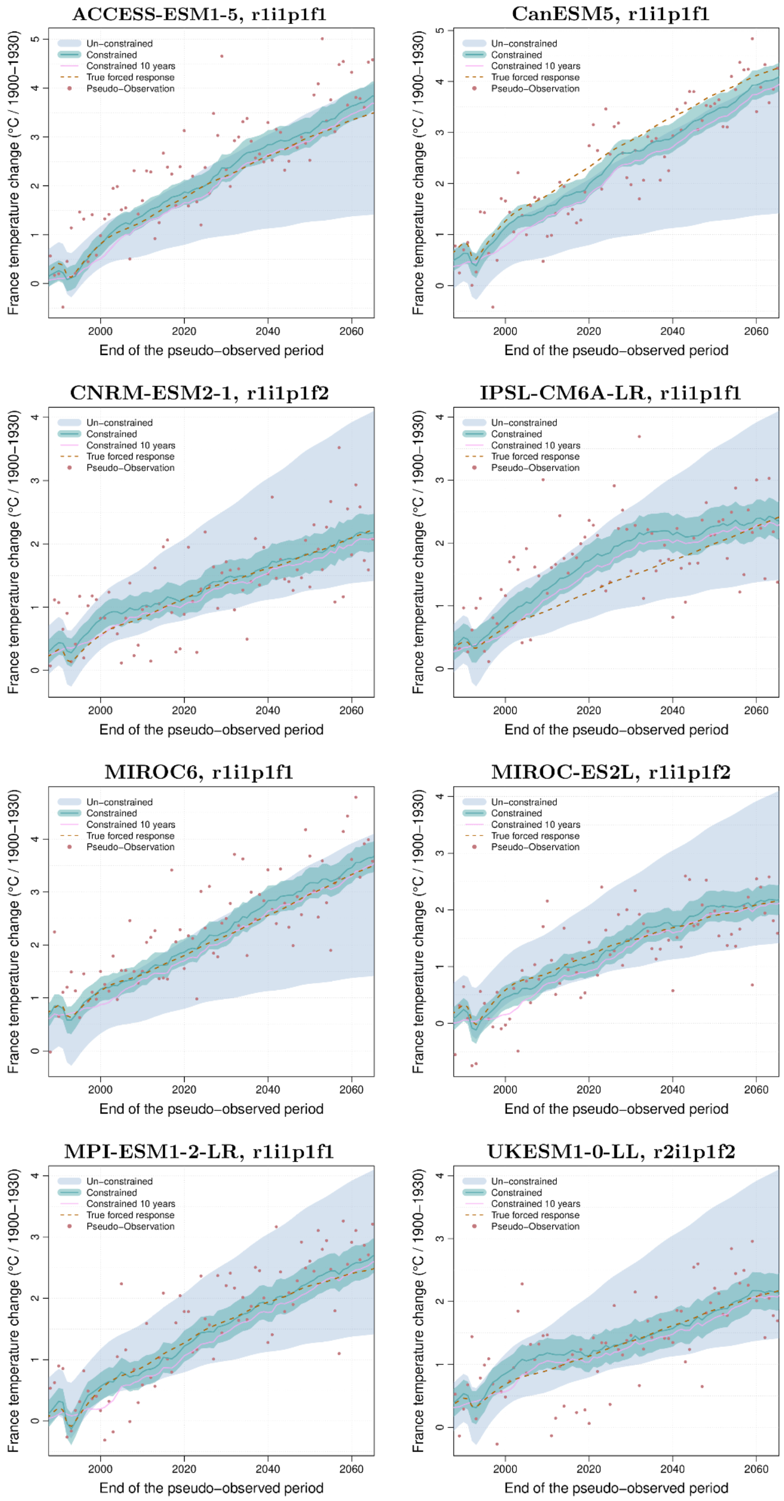

Figure S3: Same as Figure S2a, but using France temperature data from different CMIP6 models as pseudo-observations. This analysis provides an additional test of the method used to estimate the forced warming to date at the regional scale. It illustrates the properties of the 1-yr and 10-yr estimators, as compared to the estimated *true* forced response.

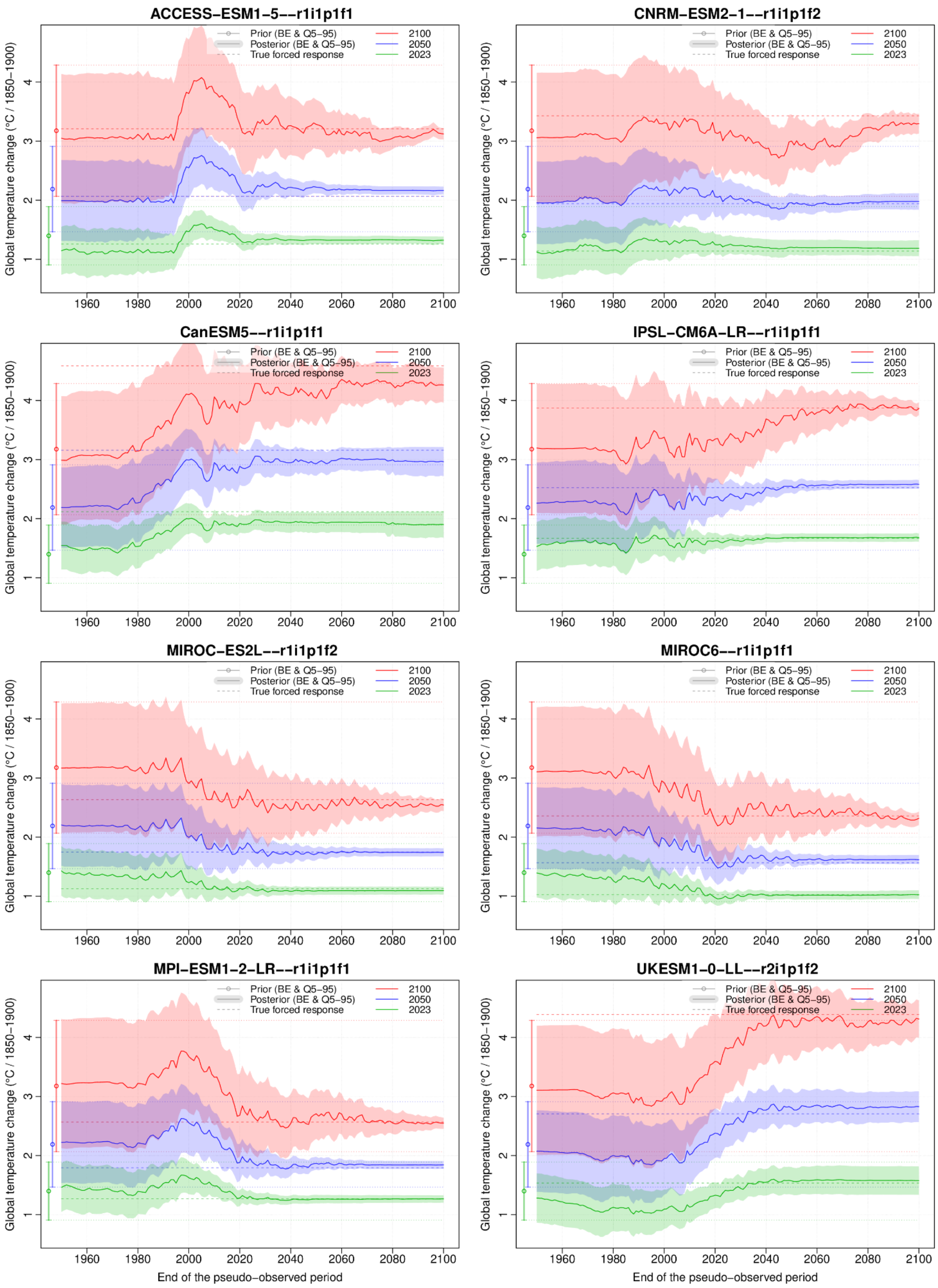

Figure S4: Same as Figure 3a, but using GST data from different CMIP6 models as pseudo-observations. This analysis provides an additional test of the method, and illustrates how constrained warming ranges improve over time and are getting closer to the estimated *true* forced response.

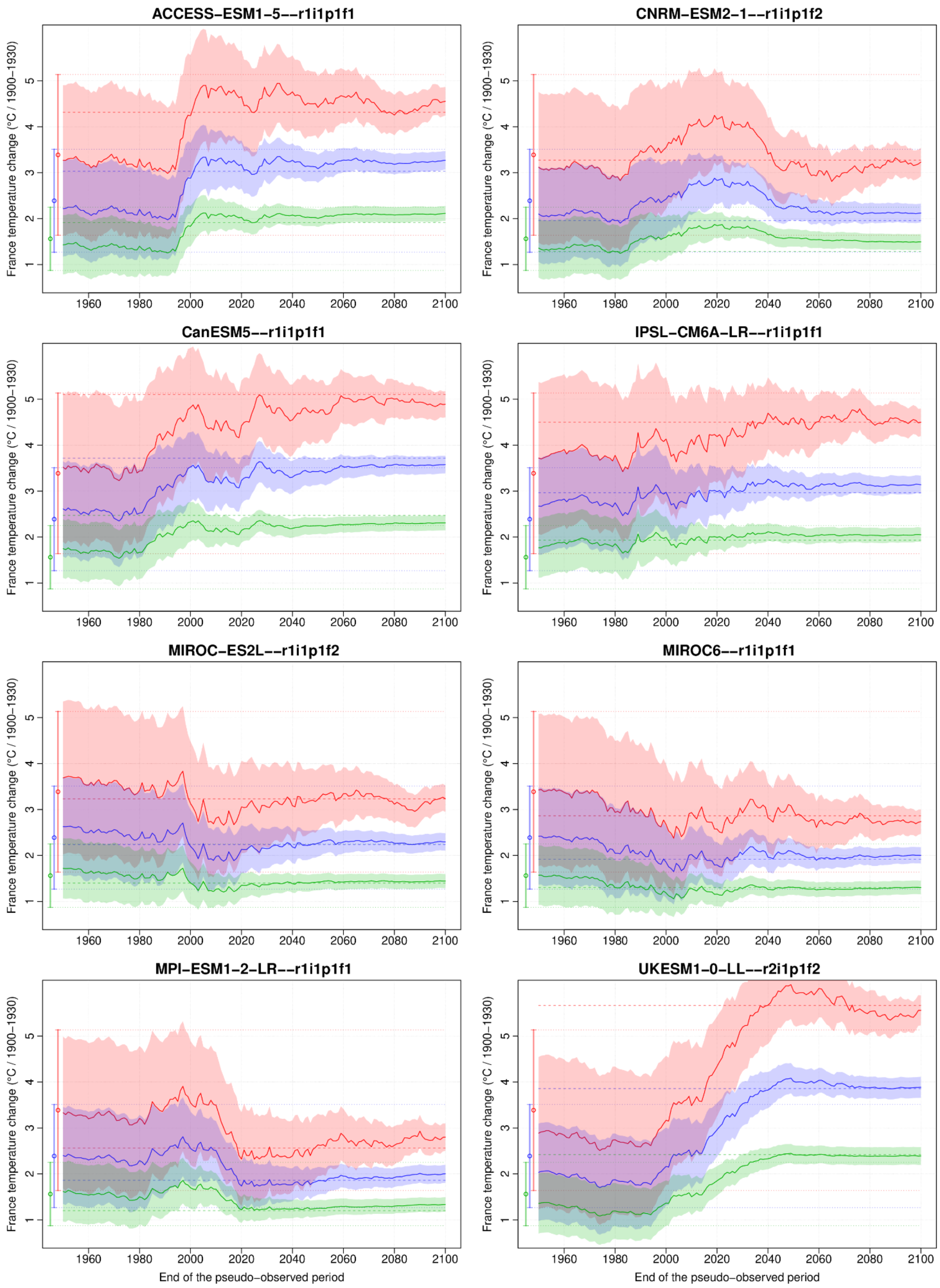

Figure S5: Same as Figure 3c, but using France temperature data from different CMIP6 models as pseudo-observations. This analysis provides an additional test of the method, and illustrates how constrained warming ranges improve over time and are getting closer to the estimated *true* forced response.
